# Hepatocellular carcinoma in adult thalassemia patients: an expert opinion based on current evidence

**DOI:** 10.1186/s12876-020-01391-z

**Published:** 2020-08-03

**Authors:** Alessandra Mangia, Davide Bellini, Umberto Cillo, Andrea Laghi, Giuseppe Pelle, Vanna Maria Valori, Eugenio Caturelli

**Affiliations:** 1 Liver Unit, Department of Medical Sciences, Fondazione IRCCS “Casa Sollievo della Sofferenza”, San Giovanni Rotondo, Italy; 2 Department of Radiological Sciences, Oncology and Pathology, “SAPIENZA” University of Rome; I.C.O.T. Hospital, Latina, Italy; 3 Hepatobiliary surgery and Liver Transplant Unit, University of Padua, Padova, Italy; 4 Department of Surgical Medical Sciences and Translational Medicine, “SAPIENZA” University of Rome; Sant’Andrea University Hospital, Rome, Italy; 5 Oncology Unit, Oncohematology Department IRCCS, “Casa Sollievo della Sofferenza”, San Giovanni Rotondo, Italy; 6 Diagnostic And Interventional Radiology Department, SM Goretti Hospital, Latina, Italy; 7 Diagnostic and interventional ultrasound unit, Medical Sciences Department, “Belcolle Hospital”, Viterbo, Italy

**Keywords:** Beta-thalassemia, Hepatocellular carcinoma, HCC, Iron overload, HCV, HBV, RMI,TACE, TARE, OLT

## Abstract

Beta-thalassemia represents a heterogeneous group of haemoglobin inherited disorders, among the most common genetic diseases in the world, frequent in the Mediterranean basin. As beta-thalassemia patients’ survival has increased over time, previously unknown complications are observed with increasing frequency. Among them, an increased risk of hepatocellular carcinoma (HCC) has been registered. Our aim is to reduce inequalities in diagnosis and treatment and to offer patients univocal recommendations in any institution.

The members of the panel - gastroenterologists, radiologists, surgeons and oncologists -were selected on the basis of their publication records and expertise. Thirteen clinical questions, derived from clinical needs, and an integration of all the committee members’ suggestions, were formulated. Modified Delphi approach involving a detailed literature review and the collective judgement of experts, was applied to this work.

Thirteen statements were derived from expert opinions’ based on the current literature, on recently developed reviews and on technological advancements. Each statement is discussed in a short paragraph reporting the current key evidence. As this is an emerging issue, the number of papers on HCC in beta-thalassemia patients is limited and based on anecdotal cases rather than on randomized controlled studies. Therefore, the panel has discussed, step by step, the possible differences between beta-thalassemia and non beta-thalassemia patients. Despite the paucity of the literature, practical and concise statements were generated.

This paper offers a practical guide organized by statements describing how to manage HCC in patients with beta-thalassemia.

## Background

Beta-thalassemia represents a heterogeneous group of inherited disorders in the synthesis of haemoglobin. It concerns homozygous and double heterozygous patients with deletions in β and δβ chains genes or, in general, genetic defects of β chains in general. Beta-thalassemia is among the most common genetic diseases in the world, frequent in the Mediterranean basin. Beta-thalassemia patients present reduced or absent synthesis of beta globin’s chains, consequent anemia due to erithroblasts destruction within the bone marrow, and red cells destruction in peripheral blood, ineffective erythropoiesis and iron overload. As beta-thalassemia patients’ survival has increased over time [1–3] new previously unknown complications are observed, including an increased risk of hepatocellular carcinoma (HCC).

In addition to the factors reported in non beta-thalassemia patients, the risk of HCC development in beta-thalassemia is linked to several factors: the high risk of infections transmitted by blood transfusions, responsible of chronic liver diseases as HCV and, at lower impact, HBV; the debatable risk that blood transfusions inhibit immune-surveillance against cancer [4] and, most importantly, the peculiar risk, compared to other transfusion-dependent blood disorders, that a progressive iron overload favors neoplastic liver transformation.

In patients with transfusion dependent (TD) beta-thalassemia major (TM), iron overload is not only a consequence of blood transfusions, but also the direct effect of the increased iron absorption. By contrast, in patients with thalassemia intermedia (TI) -defined as a clinical variant of thalassemia characterized by a thalassemia phenotype of mild-moderate degree of severity- able to maintain Hb levels of 7 g/dl without regular blood transfusion (NTD), iron overload, in addition to the increased absorption, is due to ineffective erythropoiesis. Moreover, iron chelation, regular in TD beta-thalassemia, is less codified in NTD.

Accumulating in the hepatocytes, iron plays a direct role in cancer development [5, 6]. Excess of iron not carried by transferrin as in normal individuals, but detected in forms referred as labile iron, promotes O reactive formation and seriously damages lipid membranes, intracellular proteins and DNA [7]. Consequences are mutations in some “tumor suppressor genes” including p53 and in “DNA repair” genes.

In addition to these mechanisms leading to neoplastic transformation, iron overload favors fibrosis progression by stellate cells activation and by a pro-fibrogenic effect of lipid peroxidation and is also associated with immunologic changes responsible of macrophage altered function [8].

Iron overload and HCV infection represent independent risk factors for liver fibrosis progression [9] and their coexistence enormously increases the risk of cirrhosis. Thanks to the blood donors screening [10] and to the possibility to cure HCV infection using direct acting antivirals (DAA), able to induce a sustained virological response at week 12 of follow-up (SVR12) of 98% in beta-thalassemia patients with HCV infection [11] -but also safe and easy to manage-, the relative risk of HCC is expected to significantly decline over time. While risk of HCV-related HCC will diminish, the impact of iron overload in cirrhosis development will persist unless an efficient chelation program is not undertaken. Remarkably, HCC was diagnosed even in TI patients NTD [1], currently representing in Italy about 1/3 of the total thalassemia patients.

Despite the accumulation of knowledge in this field, no practical guidelines on HCC in beta-thalassemia have been published so far. Consequently, for beta-thalassemia patients, the risk of not receiving the best treatment, because of limited local expertise, exists. The aim of this work is to reduce inequalities in diagnosis and treatment of HCC and to offer beta-thalassemia patients the best shared care in any single institution.

## Main body

### Are HCC prevalence and incidence in beta-thalassemia patients higher than in non beta-thalassemia patients?

**Table:** 

*Incidence and prevalence of HCC do not differ between beta-thalassemia and non beta-thalassemia patients.*	

According to the 2019 AIOM report, 33.000 patients had HCC diagnosis in Italy between 2013 and 2018 [12]. Among beta-thalassemia patients, HCC prevalence ranged from 2.3% in Greece to 1% in Italy [1, 13]. Another study reported that HCC prevalence in male patients with beta-thalassemia is 6 times higher than in non beta-thalassemia subjects [14].

HCC incidence, standardized by age in non beta-thalassemia patients can be estimated at 10.9/100.000 P/Y for male and 3.1/100.000 P/Y for female [13]. Incidence of 1.02/100.000 P/Y was estimated in the Italian Thalassemia Registry including 5855 patients [1]. Annual incidence of 2% was reported in a cohort of 108 TM or TI under US surveillance (C) [15].

### Who are beta-thalassemia patients at risk of HCC who need surveillance?

**Table:** 

*In addition to the standard surveillance criteria in patients without beta-thalassemia, patients to be surveilled are beta-thalassemia patients (either TM or TI) with:*	
*-chronic hepatitis HBV or HCV, with or without iron overload*	
*-liver cirrhosis of any etiology, with or without iron overload, including subjects in transplantation waiting list*	
*-iron overload, regardless of the presence of cirrhosis*	

Among patients without beta-thalassemia, HCC occurs more frequently in patients with HCV or HBV hepatitis or alcohol abuse. In Italy, the number of patients with HCC is increasing, although the etiologic factors are changing. Within the ITALICA cohort HCC increased from 624 to 1130 over time [16]. A decline in the percentage of HCV related HCC from 51 to 49%, and of HBV related HCC from 3.3 to 2.1% was reported [17]. Nowadays, HCC related to HCV and HBV infections account for 50% of liver tumors whereas HCC related to non-viral etiology make up for 32.4% [18].

In beta-thalassemia patients, HCV infection with or without iron overload has been the most frequent etiology until recently. In those who received blood transfusions before 1990, HCV infection prevalence was proportional to the number of transfused units. In studies published in 2000, HCV Ab resulted positive in 85% of Italian beta-thalassemia patients [19, 20]. Active HCV infection confirmed by HCV RNA replication has been shown in 50% of HCVAb positive subjects [21]. Higher rates of anti HCV positive patients were reported up to 1992 in Greece [22].

Careful donor screening reduced HCV related transfusion risk to 0.1 per 1.000000 [23, 24]. Therefore, patients with beta-thalassemia, infected before 1990, represent the main group at risk of developing cirrhosis and HCC. As the majority of patients has been treated with IFN ± RBV in the past or with DAA since 2014, the risk of cirrhosis of viral etiology will become less relevant in the future.

#### HBV

For many years, beta-thalassemia children have been considered at risk of HBV infection. Currently, vaccination programs and blood donor screening have been shown to drastically reduce the incidence of HBV infection in Italy.

Hepatitis B virus integrates in human DNA and is associated with an intrinsic risk of HCC induction. Prevalence of HBV infection in the Mediterranean area ranges from 0.8% in Turkey [25] to 29% in Egypt [26]. In the Italian thalassemia registry, an active HBV infection was diagnosed in 5% of patients with HCC, while a past HBV infection based on anti HBcAg positive was observed in 58% of cases [1].

#### Iron overload

Hepatocytes are the main iron storage sites in the body. No biological mechanisms exist for the excretion of excess iron. Iron overload is defined as an increase in total body iron, and liver is the most affected organ. Iron overload in beta-thalassemia occurs when (a) iron storage is insufficient to bear the excess of iron derived from erythrocyte catabolism and increased absorption in TDT; (b) levels of epcidin hormon responsible of increased iron intestinal absorption are low and the amount of iron released in the blood increases leading to an excess of iron liver storage as inTI.

When the level of safe iron sequestration is exceeded, the storage protein ferritin is denatured, releasing large amounts of iron ions into the cytoplasm of the hepatocytes. Iron accumulation in the liver results in hepatocyte damage and dysfunction leading to fibrosis development. Iron overload and HCV work in synergy in promoting fibrosis progression towards liver cirrhosis [9].

Liver iron overload “per se” may be complicated by malignant transformation in the absence of viral infections [1, 27, 28]. Several main mechanisms of cancerogenesis have been hypothesized [27–30].

#### Cirrhosis

Liver cirrhosis is the main risk factor for HCC. In patients without beta-thalassemia, cohort studies demonstrated that mortality in HCC is dependent on the severity of the underlying cirrhosis, the main etiological factors being HCV and hemochromatosis [31]. Additional risk factors were the presence of an active viral infection, older age, co-morbidities including co-infections, alcohol abuse, obesity, and diabetes.

In beta-thalassemia patients, cirrhosis is reported in 20% of cases [9, 14, 32]. However, the number of cirrhotic patients with HCC in Italy is unknown. Published data in patients with beta-thalassemia and HCC do not provide details on severity of the underlying liver disease. I.

### Does HCC development in beta-thalassemia patients necessarily require the presence of liver cirrhosis?

**Table:** 

*To present knowledge, HCC development in beta-thalassemia patients does not necessarily require the presence of liver cirrhosis and in the absence of cirrhosis it is more frequently observed inTI.*	

In non beta-thalassemia patients the prevalence of HCC in non-cirrhotic population is reported at 9.6%, with an upwards trend especially in patients with NAFLD [16]*.*

In a letter published by Restivo Pantalone, only 1 out of 9 cases had cirrhosis [33]. Of an Italian cohort published in 2014 only 4 out of 60 had cirrhosis [1]. Of the 2 HCC beta-thalassemia patients reported by Makaron, none had cirrhosis [34]. Fragatou published 5 HCC cases of whom 2 had cirrhosis [32].

Seven cases with occurrence of HCC in non-cirrhotic livers have been described: two patients with TI (one of them with HCV-positivity); one patient with TM and HCV-positivity; two patients with TI (one of them with HCV-positivity); two patients with TI and negativity both for HBV and HCV [35]. All these cases presented iron overload and although episodic, confirm the possibility that iron overload alone could induce the development of HCC [1, 30].

Persistently high LIC (Liver Iron Concentration) values as a consequence of differences in monitoring schedules either in TD or in NTD patients may play a role [36].

### Is there a residual risk of HCC in HCV infected beta-thalassemia patients who attain SVR12 after antiviral therapy, or in patients with chronic HBV infection controlled by Nucleos(t) ide analogues (NUCs)?

**Table:** 

*In patients with beta-thalassemia who attain SVR12 after antiviral therapy for HCV or control of HBV on NUCs, a residual risk of HCC is related to the severity of iron overload.*	

The recent introduction of direct acting antiviral (DAA) against HCV led to extremely high SVR12 [11, 37]. In non beta-thalassemia patients, after SVR12 the risk of HCC although reduced is not zeroed [38, 39] although not different from that after Interferon. The risk is increased by severity of the underlying liver disease and presence of co-morbidities. Not achieving DAA response was associated with increased HCC risk [40]. Chronic HBV infection is safely controlled by NUCs. In non beta-thalassemia patients, the risk of HCC after Tenofovir alafenamide or entecavir is not zeroed. HCC predictors are cirrhosis or severe fibrosis, age and male gender. Patients with NUCs-induced HBsAg seroclearance, on top of complete viral suppression, have a lower risk of HCC than those only achieving complete viral suppression under prolonged NUCs treatment [41].

In patients with NUCs -induced HBsAg lost, HCC risk is similar to that of patients with spontaneous HBsAg lost and can be estimated around 1% at 5 years [42].

There are no available data in the setting of beta-thalassemia patients where the risk may be increased by the presence of iron overload.

Biannual surveillance is required in at risk patients regardless of SVR12 achievement or NUCs induced HBV DNA control.

### What is the most accurate test to non-invasively diagnose advanced liver fibrosis in beta-thalassemia patients?

**Table:** 

*Transient elastography is the most accurate non-invasive test for assessing advanced liver fibrosis in beta-thalassemia patients.*	

In patients with beta-thalassemia the evaluation of LIC by MR Imaging has replaced iron concentration in mg/g of dry liver on liver biopsy. In parallel, formerly invasive diagnosis of liver damage can be now performed non-invasively evaluating liver stiffness by transient elastography (TE). In not beta-thalassemia patients, TE is the most accurate non-invasive assay in excluding cirrhosis (AUROC 0.90) [43]. The AUROC for fibrosis comparable to a histological stage ≥2 is 0.86 [44, 45].

In patients with beta-thalassemia, this method was evaluated in 4 studies. The first is a controlled cohort study including 56 patients who underwent both liver histology and TE. Results showed that TE is able to discriminate between advanced fibrosis and liver cirrhosis and that the presence of iron overload is not a confounder [45].

A second study was “cross sectional” and included 115 patients with TM or TI. However, only in 14 of them a direct comparison between TE and liver histology was possible [46]. In patients with positive HCV RNA and ferritin levels > 1000 ng/ml, TE overestimated liver fibrosis.

The third study based on 15 patients who underwent both liver biopsy and TE, demonstrated that TE is accurate in assessing the presence of significant fibrosis in patients with beta-thalassemia, showing a sensitivity of 60% and a specificity of 89%. However, the highest accuracy was attained in diagnosis of cirrhosis with a sensitivity of 100% and a specificity of 92% [47]. The fourth study on 49 patients without HCV infection who underwent T2*MR and TE showed a significant direct correlation between iron accumulation and liver stiffness [48]. In conclusions, in the absence of an active HCV infection, TE is as accurate in determining advanced fibrosis status as in patients without beta-thalassemia.

### Is the recall policy for early detection of HCC in beta-thalassemia patients different from that used in non beta-thalassemia ones?

**Table:** 

*In beta-thalassemia patients, surveillance for early detection of HCC should be based on abdominal US and serum AFP determination every 6 months and on periodic MRI performed according to baseline LIC.*	

In beta-thalassemia patients, surveillance is needed either to determine iron accumulation into the liver, or to early detect possible HCC growth.

Measurement of iron concentration in liver biopsy (LIC), representative of total body iron stores, has been replaced by MRI LIC assessment [49].

The current surveillance program suggested for beta-thalassemia patients [50], is stratified according to NTDT or TDT condition and baseline LIC. Both NTDT and TDT patients must undergo biannual MRI if baseline LIC is > 15 mg/g. If baseline LIC ranges between 5 and 15 mg/g (NTDT), and between 3 and 15 mg/g (TDT), MRI must be performed annually.

When baseline LIC is < 5 mg/g (NTDT) or 3 mg/g (TDT), MRI is advised every 2 years.

Optimal frequency for HCC surveillance is every 6 months (semi-annual). The observed improvement of overall survival in a large Italian cohort [17] is also owed to the wider use of semi-annual surveillance, expanding the proportion of HCC susceptible of curative treatments. Another study in the same cohort [51, 52], had previously demonstrated that semi-annual US surveillance has a positive factual benefit in a long-term perspective, not due to lead-time bias, compared to annual one.

Also in beta-thalassemia patients at risk of HCC, surveillance should be performed by abdominal US and AFP serum determination every 6 moths [33, 53]. As for viral infection monitoring, patients undergoing blood transfusion should be tested for HCV Abs and HBsAg every year.

#### AFP

Although AFP is increased > 400 ng/ml (the value considered diagnostic for HCC) in only 20–25% of patients with HCC, either with beta-thalassemia [1, 33] or without [54, 55], it allows to identify patients at risk when baseline values are > 20 ng/ml, or if they increase over time [56]. Increased AFP is associated with an unfavorable outcome [57]. When increased at baseline, it is of great value in monitoring patient treatment response [57, 58].

#### Ultrasound

US surveillance is essential in detecting small lesions susceptible of curative treatment [17, 51]. For surveillance of HCC in patients without beta-thalassemia, US has an excellent specificity (90%) and a sensitivity ranging from 58 to 89%, so often allowing a curative treatment [17, 59].

US sensitivity in the diagnosis of early (< 2 cm) HCC is not higher than 63%, being such a technique dependent on both operator experience [60] and equipment quality [61]. Limitations to optimal performance of US scanning are obesity, meteorism, presence of coarse-echo-texture in cirrhotics, and low level of patient’s compliance.

There are no studies available on the efficacy of US surveillance programs in beta-thalassemia patients, but there are no reasons to hypothesize differences in diagnostic accuracy as compared to non beta-thalassemia patients.

#### MRI

In patients affected by beta-thalassemia, MRI with hepatobiliary contrast agent is the best imaging method for confirming the diagnosis of HCC. This because MRI is also sensitive to the presence of iron within tissues and allows quantification of iron overload in the liver. MRI must be performed routinely in beta-thalassemia patients beyond the specific context of HCC surveillance, since it is essential to determine the entity of iron overload in the liver.

A quantification of LIC is necessary to establish the risk of HCC and the frequency of surveillance. Using MRI, the precise quantification of the iron overload can be performed by two complementary methods validated in the literature. The liver to muscle ratio or SIR (signal intensity ratio); and the calculation of T2* or R2* (=1/T2*) and its conversion to LIC. Both methods have advantages and disadvantages. Fortunately, there are free software that combine both methods allowing a more precise iron quantification. The MRQuantif software takes into consideration the various T2* values, the quality of the fit of the curve with the points for each T2*, the steatosis quantification and the SIR result to determine the optimal T2*.

Should MRI not be available, serum ferritin level may be used as a surrogate in TDT patients, being simpler and cheaper than MRI. In NTDT patients, serum ferritin level may underestimate liver iron overload, since increased iron absorption characterizing such condition leads to intracellular storage, with lower levels of serum ferritin.

Regarding HCC diagnosis, after a suspicion of HCC raised by US surveillance, a confirmation imaging technique must be performed. According to the most recently published EASL Guidelines on Management of HCC, CT or MRI should be used at this purpose because of their higher sensitivity. Three meta-analysis [62, 63] underlined the superior value of MRI over CT, especially in small lesions and if a hepato-specific contrast medium was used. CEUS should be considered a potential alternative, but lacking strong evidence it cannot be recommended. In comparison to CT or MRI, CEUS sensitivity is significantly lower, especially in nodules of 10 to 20 mm, because of a lower detection rate of washout [63, 64].

None of the previously cited diagnostic procedures is contraindicated in beta-thalassemia patients.

Apart from the usual differential diagnoses, in beta-thalassemia patients extramedullary hematopoiesis (EMH), a relatively frequent feature in inefficiently transfused patients with TM and much more in patients with TI, should be differentiated from hypovascular HCC. In these cases characterization might require biopsy. EMH may occur in different sites of the body with the most frequent being paravertebral areas, liver, spleen and lymph nodes [65]. Liver involvement is usually diffuse, resulting in hepatomegaly. Focal masses within the liver are much less common, and only around ten of such cases have been reported from 1980 to 2000 [66]. These mass-like foci of EMH need to be distinguished from neoplasms.

On imaging, EMH lesions are well demarcated and usually appear hypoechoic on ultrasound and hypodense on CT, whereas they are hypointense in T1 and hyperintense in T2 on MRI [67].

Although these lesions do not present typical imaging features of HCC, they might create difficulties in differential diagnosis with hypovascular HCC. So, usually these tumor-like lesions require image-guided biopsy for definitive diagnosis [67].

### Is the management of early HCC the same in beta-thalassemia and non beta-thalassemia patients?

**Table:** 

*A multidisciplinary approach to individualize treatment of early HCC in beta-thalassemia patients should be used.*	

According to the current guidelines on HCC diagnosis and treatment [68], “early HCC” is defined into two different stages (“very early”, or stage 0, and “early”, or stage A) according to liver function, performance status (ECOG-PS), and tumor burden (Table 1).

**Table 1:** Stage classification of patients with HCC

***Stage***	*Very Early Stage* *0*	*Early Stage* *A*	*Intermediate Stage* *B*	*Advanced* *Stage* *C*	*Terminal Stage* *D*
***Liver Function***	*+*	*+*	*+*	*+*	*–*
***Performance Status***	*ECOG-PS* *0*	*ECOG-PS* *0*	*ECOG-PS* *0*	*ECOG-PS* *1–2*	*ECOG-PS* *1–2*
***Tumor Burden***	*Solitary nodule ≤ 2 cm*	*Solitary nodule ≤ 2 cm .* *2–3 nodules all < 3 cm*	*Multinodular (> 3 nodules, or ≥ 2 nodules if any > 3 cm)*	*Macrovascular invasion or extraepatic spread*	*Non-transplantable HCC*

ECOG-PS (esternal cooperative oncology group performance status) [69]

Subject to liver function preserved (Child-Pugh score A), and ECOG-PS 0 in both groups, “very early stage” is defined when the tumor burden is represented by a solitary nodule ≤2 cm in diameter, and “early stage” when a solitary nodule is > 2 cm in diameter or up to 3 nodules are all ≤3 cm in diameter.

Though Asian [70], American [71], and European [68] guidelines still recommend ablation only in patients with stage 0 and A who are not candidates for resection, such technique is now widely considered the first option in solitary tumors ≤2 cm in diameter favorably located within the liver. The most used method is US-guided percutaneous radiofrequency ablation, which causes tumor necrosis by thermal damage [72]. The success rate is inversely related with nodule diameter, so resulting in a worse local control for tumors > 3 cm. However, for nodules up to 3 cm, thermal ablation has been demonstrated equivalent to resection in terms of cumulative recurrence rates and overall survival [73] and is burdened by fewer complications. On the other side, resected patients present higher rates of perioperative mortality and major complications, but also lower rates of local tumor progression [74].

Among ablative therapies, percutaneous ethanol injection (PEI) has been substantially replaced by thermal ablation in the treatment of HCC < 2 cm because of its higher rates in cumulative recurrence and local tumor progression, in spite of similar 5-year overall survival [75]. Moreover, the extension of tumoral necrosis produced by PEI is scarcely predictable. Very recently, microwave ablation is achieving a wider role as compared to RF, thanks to its capacity (due to greater usable powers) to obtain similar results in shorter times.

Resection, in stage 0, is preferred to thermal ablation in selected cases, above all when a laparoscopic approach is feasible, or when the tumor site is not reachable by percutaneous ablative techniques.

In stage A, when the tumor is solitary, resection is the first option regardless of tumor size, above all when there is no clinically significant portal hypertension [76]. If surgical resection is not possible, liver transplantation must be considered.

For beta-thalassemia subjects with early HCC, data available in the literature are scanty, heterogeneous and tailored to individual patients’ needs. In an Italian cohort study published in 2014 [1], HCC was already advanced at the time of diagnosis in 21 out of 62 cases (33.9%), so that only palliative treatment was possible. US surveillance is essential in detecting small lesions susceptible of curative treatment [17].

There are no sufficient data to establish which is the best therapeutic approach in early HCC among thermoablation, liver resection, and liver transplantation. There are no data suggesting that the management of early HCC should be different in patients with or without beta-thalassemia [76]. A multidisciplinary approach involving hematologists, hepatologists, radiologists and oncologists is essential in identifying a tailored treatment [77].

### In beta-thalassemia patients with HCC are liver resection indications different from those in patients without beta-thalassemia?

**Table:** 

*Surgical treatment of HCC in patients with beta-thalassemia, should follow the same indications applied in non beta-thalassemia patients; because of the higher risk of thromboembolic events during the early postoperative course aggressive coagulation prophylaxis should be adopted.*	

In latest years, only a few studies have evaluated the surgical treatment of HCC in patients with beta-thalassemia [77, 78].

Beta-thalassemia patients with HCC present relevant co-morbidities: hypogonadism (55%), myocardiopathy (52%), hypothyroidism (42%), osteoporosis (31%), diabetes (31%) and chronic renal failure (4%) [79]. This needs to be taken into account when planning surgical resection.

In the largest available study [1] the same therapies currently adopted in non thalassemia patients, either loco-regional, or resective, or chemotherapeutic, or palliative were adopted for beta-thalassemia patients. Such approach, even when aggressive as liver resection, led to a survival improvement in beta-thalassemia patients with HCC (median of 11.5 months) as compared to 2004 (3.5 months median time from diagnosis to death) [1].

Both Maakaron [34], who described a recurrent HCC treated by liver lobectomy after percutaneous thermal ablation, and Moukhadder [35] stated that both surgical and loco-regional ablative therapies should be considered for the treatment of HCC in beta-thalassemia patients. They appear to be safe and effective options [1, 16, 77].

The adoption of a more invasive treatment, albeit potentially radical as surgical resection, is also supported by the scarce availability of relevant alternatives. Obviously, an extensive evaluation of potential co-morbidities is strongly suggested in the preoperative workup.

Even though there might be no need for specific cardiologic evaluations, it is advised to perform, in patients undergoing surgery, an extremely accurate workout for pulmonary hypertension (PH) that seems more common in adult patients with TI not or inefficiently transfused. Indeed, it is well shown how precapillary PH may be a devastating complication in beta-thalassemia [80]. An echocardiogram and an echo-stress test may be suggested in all patients in order to exclude subclinical heart failure (HF) [81].

Pulmonary atelectasia has been frequently described in the setting of beta-thalassemia patients undergoing splenectomy. A preoperative spirometry may be indicated in patients with other risk factors for postoperative ventilatory dysfunction [80].

Present evidence on the best surgical approach between laparotomy and laparoscopy in the setting of HCC are currently limited. However, studies have been published in the particular setting of splenectomy in beta-thalassemia patients [81, 82]. A single randomized study comparing laparotomic vs. laparoscopic splenectomy showed an increased incidence of intraoperative and postoperative bleedings in the laparoscopic group [83]. The results, however, did not reach statistically significant difference [83].

On the other hand, in the HCC general population, laparoscopic approach has been found to be significantly beneficial in terms of postoperative morbidity, mortality, length of hospital stay and blood transfusion requirement when compared to the open approach [81]. On these bases, we suggest that HCC surgical approach in beta-thalassemia patients should be adopted in tertiary surgical centers with high volume of both open and laparoscopic procedures where the laparoscopic choice can be considered in every case and adopted safely.

As far as the postoperative course is concerned, it has to be underlined that thrombotic risk is increased in beta-thalassemia patients. Few recent evidence report a higher risk of thromboembolic events in particular during the early postoperative course [84, 85]. However, no clear evidence supporting thromboembolic prophylaxis different from non thalassemia patients is currently available.

Even though studies specifically conducted in beta-thalassemia patients undergoing liver resection or transplantation for HCC are not available to date, close postoperative surveillance and aggressive coagulation prophylaxis should be adopted in these patients.

**Table:** 

*Based on the limited evidence available, liver transplantation in beta-thalassemia patients is not associated with higher morbidity or mortality compared to patients who are usually referred to liver transplant. Beta-thalassemia should not be considered per se as a contraindication to liver transplantation.*	

### In beta-thalassemia patients with HCC is liver transplantation associated with higher perioperative mortality?

Liver Transplantation (LT) is now considered the treatment of choice in patients with HCC. In the past, mainly due to cardiac co-morbidities [77], LT has long been refused to beta-thalassemia patients with end-stage liver disease or HCC. Beta-thalassemia is not longer considered a contraindication to liver transplant anymore provided that severe PH and subclinical HF are excluded [86]. Thanks to better chelation therapy and iron overload monitoring [1] leading to improved survival, both liver failure and HCC have become a more commonly reported complication, with significant adverse impact on prognosis and mortality [1].

In the series of nine beta-thalassemia patients with HCCs reported by Restivo Pantalone, four patients died during follow-up due to decompensated cirrhosis. The single patient undergone LT (after thermal ablation and TACE) survived 69 months, compared to a median survival of 25 months for four not transplanted patients. The authors concluded that beta-thalassemia should not be considered a contraindication for either treating HCC or for LT [33].

Mancuso described two additional patients undergoing successful LT with satisfactory post-transplantation outcomes, without severe complications after 6 months and 2 years of follow-up, respectively [77].

Borgna-Pignatti described three patients undergoing LT, two of whom died shortly afterwards for reasons unrelated to beta-thalassemia [1]. Therefore, after excluding significant co-morbidities as heart dysfunction and PH, beta-thalassemia should no longer be considered a contraindication to liver transplantation which is, as in non beta-thalassemia patients, a potential therapeutic option conferring better survival [85, 86].

As reported, only six patients underwent LT for HCC, but the outcomes were affected by beta-thalassemia complications [33].

As, HCC and liver failure can develop in young beta-thalassemia patients: in this context, the relevant survival benefit offered by LT compared to the standard alternative treatments (transplant benefit) has to be considered.

Prevalence of HLA antibodies is relevantly increased in TM. Even though a high panel reactive antibody percentage is not a contraindication to transplant, the possibility of antibody mediated events has to be carefully considered in postoperative course. On these bases, a regular evaluation of circulating Donor Specific Anti HLA antibodies (DSA) has to be adopted in beta-thalassemia patients undergoing LT [87].

### In beta-thalassemia patients with cardiac dysfunction associated with iron overload, is a combined heart and liver transplantation an option to be considered?

**Table:** 

*In highly selected patients, combined heart–liver transplantation could be considered as a possible therapeutic option.*	

Current indications for combined heart–liver transplantation (CHLT) include end-stage heart and liver disease of varying etiology, in particular familial amyloid polyneuropathy and heart failure with associated cardiac cirrhosis. Liver iron overload leads progressively to chronic liver damage, and some patients may develop liver cirrhosis associated to heart failure.

To date, only one combined heart and liver transplantation has been reported in the setting of beta-thalassemia [88]. The patient, with a homozygous beta-thalassemia, was diagnosed when he was 2 years old and started blood transfusions when he was four. At age 11 he underwent splenectomy in order to maintain adequate level of hemoglobin. He started deferoxamine therapy but with low compliance. At age 17 he began to show signs and symptoms of cardiac failure due to heavy iron deposition, confirmed with MRI. The left ventricular ejection fraction was 21%. Despite patient’s improved compliance to the therapy, sign of liver failure started to rise: prolonged prothrombin time, low albumin level, increase in liver enzymes. Eventually, he developed ascites and a liver biopsy showed heavy iron loading and portal fibrosis with cirrhosis. At age 26 he underwent combined cardiac and liver transplantation. Eighteen months after transplantation liver showed only focal iron deposition and mild rejection. At a 2-year follow-up, the patient’s hepatic and cardiac functions were normal. The authors concluded that they would consider combined organ transplantation for patients with iron loading and severe cardiac dysfunction associated with histological diagnosis of cirrhosis. This may be the only option for patients with end stage iron -related cardiac and liver disease [88].

### Are the therapeutic efficacy and complications of TACE and TARE in beta-thalassemia patients the same as in the non beta-thalassemia patients?

**Table:** 

*Despite the lack of specific evidence on the use of TACE or TARE in the treatment of HCC, patients with beta-thalassemia should follow the same clinical indications applied to non beta-thalassemia patients.*	

Despite the absence of specific studies evaluating TACE in the treatment of HCC in this specific patient’s population, important data can be derived from numerous studies demonstrating efficacy and safety of this procedure in HCC treatment of non beta-thalassemia patients.

According to the Barcelona clinic Liver Cancer (BCLC) system, the most commonly used staging system for treatment and prognosis of HCC in Western countries [89], TACE is the only recommended treatment strategy in intermediate stage (BCLC B). However, not all patients benefit from TACE in the same way [90] and such heterogeneity has prompted authors to identify prognostic parameters and different scores enabling patients’ stratification.

Bolondi and coworkers [91] proposed a sub-classification which identified four sub-stages (B1–B4) of intermediate HCC, incorporating the new concept of joint consideration of the tumor burden according to the “beyond Milan” and the “within up-to-7” criteria together with the Child-Pugh score and PS.

For B1 patients, considering their conserved liver function and limited tumor burden, TACE is suggested as first option. For B2 patients [92], 90Y-radioembolization (TARE) or sorafenib could be considered in cases expected not to respond to, to be poor responders or to have contraindications to conventional TACE. For the substage B3, the option of new treatments tested within clinical trials represents the only therapeutic alternative to sorafenib or TACE. In substage B4 patients who met the up-to-7 criteria, liver transplantation is the best option. In patients without alternative therapeutical options, symptomatic treatment to avoid unnecessary suffering from liver damage can be offered [93].

New recent evidence suggest the possibility to successfully and safely combine TACE with sorafenib in non beta-thalassemia patients [94].

Trans-arterial radioembolization (TARE), consisting of selective internal radiation therapy (SIRT), is a well-recognized therapy in a number of guidelines on clinical management of non-resectable HCC [95]. In patients in intermediate stages and history of prior TACE failure or tumoral macrovascular invasion in the absence of extra-hepatic spread, according to ESMO (European Society of Medical Oncology) guidelines, TARE may compete with sorafenib [96].

From a technical point of view, TARE is a catheter-based interventional procedure allowing the emission of β-radiations at therapeutic levels into the tumor through its feeding arteries. Such ultra-selective approach, as for TACE, is aimed to minimizing the effect on healthy liver parenchyma close to the tumor. The local brachytherapy performed by TARE, conversely to TACE, doesn’t result in tumor ischemia due to microvascular embolization [97]. Devices for radioembolization are commercially available in form of implantable glass (Therasphere®) or biocompatible resin-based (SIR-Spheres®) radioactive (Yttrium^90^ - Y^90^) spheres [97].

TARE is safe [98], also compared to conventional Sorafenib treatment, and no particular attention should be paid in beta-thalassemia patients. Usually, after the procedure, oral amoxicillin/clavulanic acid (375 mg 3 times per day) and pantoprazole (40 mg per day) are administered for 3 days [99]. Discharge from the hospital is decided according to individual patients’ clinical status. Clinical and laboratory follow-up is advisable [99].

In recent years, the radial approach has been introduced. This technique guarantees a safe vascular access, especially if compared with the brachial one, and allows patients to spend a more comfortable post-operative period considering the possibility of walking immediately after the procedure. Thanks to a greater efficacy of the hemostasis means, this approach is also advisable in patients with coagulation alterations [99].

### Which is the systemic therapy and the level of safety in the treatment of advanced HCC in beta-thalassemia patients?

**Table:** 

*In case of advanced HCC, first-, second- and later-line therapy or the use of checkpoint Inhibitors should follow the same indications of non beta-thalassemia patients. In the absence of specific contraindications, the safety profile of approved drugs appears comparable in patients with or without beta-thalassemia.*	

#### First line therapy

**Fig. 1:**
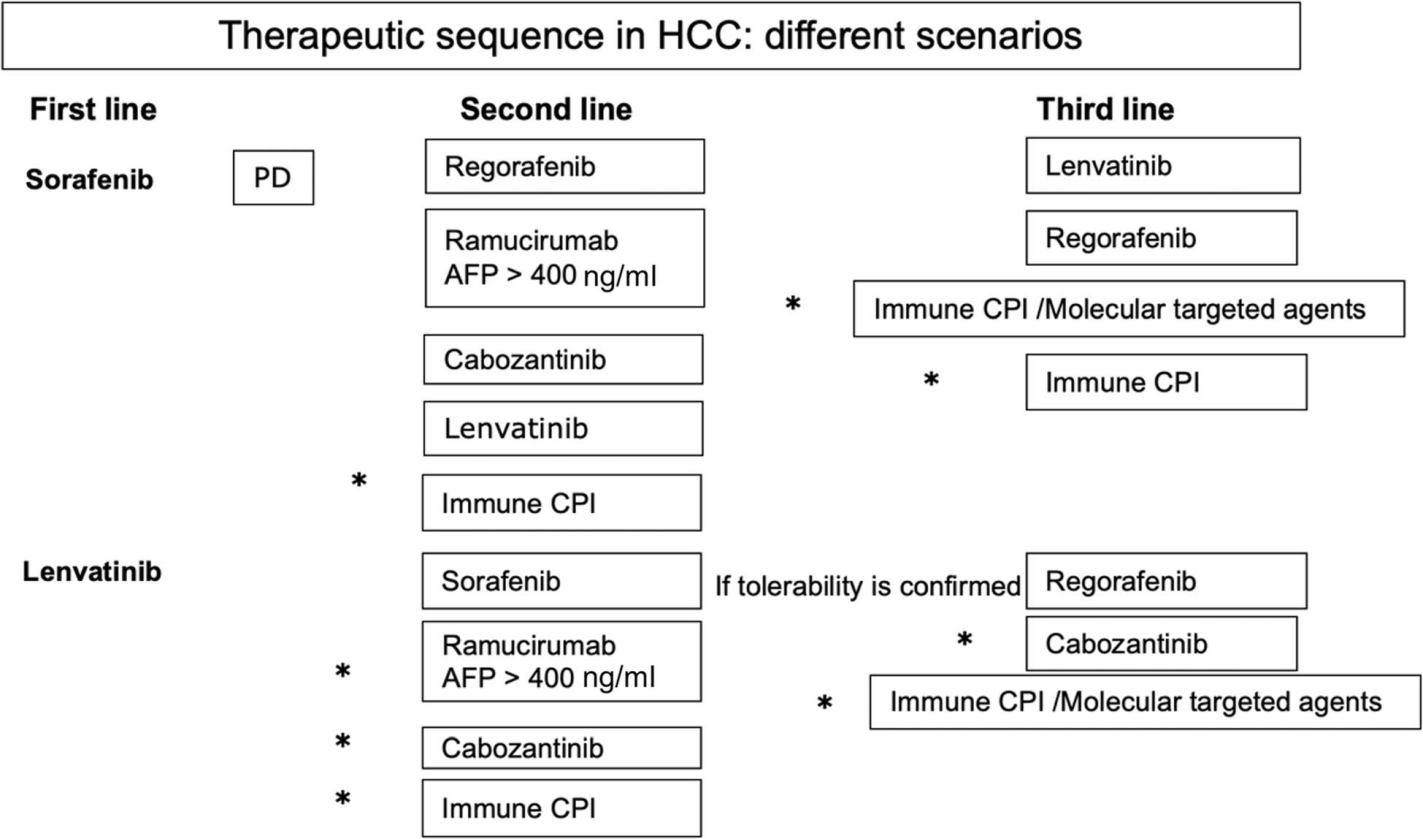
Therapeutic sequence in HCC: different scenarios. *Asterisks indicate that therapeutical sequences are possible but not yet documented in the literature. Use of regorafenib, ramucirumab and cabozantinib or lenvatinib after sorafenib has been proven to be effective. The role of Immune CPI alone or in combination with molecular targeted agents are still under investigations*

Recent progress in the area of systemic therapy for advanced HCC in non beta-thalassemia patients have improved life expectancy (Fig. 1). Sorafenib (400 mg daily) or Lenvatinib (8 mg daily), are current first line drugs, and provide similar survival benefit in patients with advanced HCC.

The multiple tyrosine kinase inhibitor (TKI) Sorafenib, approved in 2007, based on results from the SHARP trial [100] improved the overall survival (OS) versus placebo (median 10.7 vs. 7.9 months, respectively) in 602 patients with advanced HCC (Child-Pugh A) previously untreated with systemic therapy [101]. The median time to radiologic progression (TTP) was significantly longer with sorafenib (5.5 vs. 2.8 months with placebo). Treatment-related adverse events occurred in 80% of the patients, diarrhea and hand-foot skin reaction being the most frequent. In case of such adverse events, patients may take advantage of symptomatic medications, although t treatment discontinuation may be required in some cases. Clinically symptomatic vascular disease (either coronary or peripheral) represent formal contraindication [102].

Based on results from REFLECT trials, levantinib was approved in 2019 [103]. Levantinib is an oral multikinase inhibitor targeting VEGFR1–3 and fibroblast growth factor receptor (FGFR) 1–4; it. was shown non inferior to sorafenib in the above open-label, phase III trial enrolling patients with advanced HCC (with the exclusion of patients with main portal vein or bile duct invasion and > 50% of liver tumor burden). Levantinib doses need to be adjusted by body weight. Incidence of hand-food skin reaction is lower than that reported with sorafenib, although higher incidence of hypertension, proteinuria and anorexia has been reported. Due to lack of solid evidence, the choice of treatment and the sequence of first-, second-, and later-line treatments for advanced HCC in beta-thalassemia patients remain complicated. Only 3 cases of beta-thalassemia patients treated with first line sorafenib are currently reported, and their treatment outcomes are unclear [1]. No data on lenvatinib treatment are available in beta-thalassemia.

#### Second- and later-line therapy

For patients who fail or were not able to tolerate the first-line, second- and later-line treatments are needed. From 2009, most clinical trials for second-line agents failed to show any improvement in outcomes among non beta-thalassemia -prior sorafenib-treated- patients. Second line therapy should not be different in beta-thalassemia as compared to non beta-thalassemia patients. Safety profile appears also comparable in the absence of specific contraindications, but no data are available in beta-thalassemia settings.

Only recently, 3 new drugs were shown effective. Regorafenib**,** an oral multikinase inhibitor, based on result from RESORCE trials [104], approved in 2019 for second line, at dose of 160 mg daily represents the current standard of care for patients with advanced HCC progressing under sorafenib. Regorafenib is recommended in patients with well-preserved liver function and ECOG PS 0–1. The phase III study, comparing regorafenib with placebo in patients with progression despite sorafenib, has reported mean OS 7.8–10.6 months [104]. Treatment improved survival in both BCLC C, and in BCLC B patients. Regorafenib seems to be better tolerated than sorafenib.

For patients in second-line treatment with baseline AFP ≥400 ng/ml, well preserved liver function and ECOG PS 0–1, ramucirumab (RAM) can be considered a valid therapeutical option, pending EMA approval [103]. RAM is a human immunoglobulin G1 (IgG1) monoclonal antibody (mAb) that inhibits ligand activation of VEGFR2. In the REACH trial, mean OS in the overall population was not statistically higher. Only the subgroup of patients with AFP ≥400 ng/mL had an improvement. Hypertension and hyponatremia were frequently reported [105].

In patients with progressive disease on one or two systemic therapies, well-preserved liver function and ECOG PS 0–1-pending EMA approval- cabozatinib represents a therapeutic option (Table 1). Phase III CELESTIAL trial [106] evaluated cabozantinib versus placebo in 707 Child-Pugh A patients with HCC progression despite 2 prior systemic regimens. Treatment with cabozantinib resulted in a very limited prolonged OS than placebo (mean OS of 10.2 months with cabozantinib and 8.0 months with placebo). Moreover, the rate of high-grade adverse events in the cabozantinib group was approximately twice that of placebo. In the absence of comparative data between these three agents, ramucirumab may be considered the best choice for patients with AFP ≥400 ng/mL in the event of approval [107]. While well tolerated in the REACH-2 trial, careful monitoring for hypertension, as for cabozantinib and regorafenib, is required.

Definite conclusions cannot be currently drawn [108] and longer follow-up periods will be necessary to understand efficacy and safety of these three new drugs in non beta-thalassemia patients. It is reasonable that beta-thalassemia patients with HCC should be managed similarly to their non beta-thalassemia counterparts.

#### Checkpoint inhibitors

The idea of activating the immune system to target the tumor, rather than directly affecting the cancer cell is not recent. This approach represents a change in therapeutic paradigm, although there are no specific studies performed in patients with beta-thalassemia. The PD-1 receptor (Programmed Death 1) is an inhibitory receptor expressed on the surface of T lymphocytes. Nivolumab is an all-human anti-PD-1 monoclonal antibody capable of inhibiting this immune checkpoint and is currently being studied in a broad clinical development program in different types of cancer, alone or in combination with other therapies [109]. The study with CPI in HCC (Checkmate-459 Nivolumab vs sorafenib) is closed, but unfortunately the expectations were not met.

### Is it possible to plan a therapeutic sequence in beta-thalassemia patients with HCC?

**Table:** 

*In beta-thalassemia patients with HCC, planning a therapeutic sequence may be even more important than in not beta-thalassemia. Due to the high frequency of splenectomy or co-morbidities, a personalized approach by a multidisciplinary group is essential.*	

Treatment options for HCC in beta-thalassemia patients are largely based on data extrapolated from general population due to the low incidence of HCC in beta-thalassemia. Beside the issue of limited number of beta-thalassemia patients with HCC treated with TKI, the best treatment choice remains controversial. Even more than in other cancers, it is essential that the most appropriate treatment is administered at the right time, considering that suspending therapies might be harmful to the patient. Furthermore, depriving patients of a possible therapeutic alternative available today exposes them to worsening of the liver function that could make an effective alternative treatment no longer feasible.

For these reasons, it is essential that since first diagnosis, beta-thalassemia patients with HCC are taken over by a multidisciplinary group, following them throughout their therapeutic path [107]. The value of a similar approach is increased in the presence of co-morbidities, possible drug interactions (especially hormonal), or metabolic diseases [110]. A number of beta-thalassemia patients has undergone splenectomy and requires medium and long-term prophylaxis protocol with all the scheduled vaccination calls, before starting cancer treatment. Particularly important are C-conjugated or tetravalent anti-meningococcal vaccination and anti-pneumococcal vaccination.

As in non beta-thalassemia patients, a clear therapeutic hierarchy should be adopted in real life in beta-thalassemia patients as well. It will explore, at first, indication and feasibility of potential radical options as liver resection, ablation or liver transplantation. If radical approaches are not indicated, embolo-therapies represent the second choice followed by first and second line systemic therapies to be adopted according to the multidisciplinary team decision.

## Conclusions

A homogeneous management of HCC in beta-thalassemia patients is a need for patients and beta-thalassemia center specialists alike. Despite a gap, due to the paucity of high-quality studies with adequate sample size to determine preferences, there are specific situations -mostly related to the iron overload- that assist in differentiating the diagnostic and therapeutical algorithms in this setting.

## Data Availability

Not applicable.

## Abbreviations

HCC: Hepatocellular carcinoma
TACE: Trans-arterial chemoembolization
HBV: Hepatitis B virus
HCV: Hepatitis C virus
RMI: Magnetic Resonance Imaging
TARE: Trans-arterial redioembolization
OLT: Orthotopic liver transplantation
